# Muscle-derived exosomes and exercise in cancer prevention

**DOI:** 10.3389/fmmed.2023.1202190

**Published:** 2023-06-06

**Authors:** Daniela Vitucci, Domenico Martone, Andreina Alfieri, Pasqualina Buono

**Affiliations:** ^1^ Department of Movement Sciences and Wellbeing, University Parthenope, Naples, Italy; ^2^ CEINGE-Biotecnologie Avanzate Franco Salvatore, Naples, Italy; ^3^ Department of Economics, Law, Cybersecurity and Sport Sciences—University Parthenope, Naples, Italy

**Keywords:** exercise, cancer, exosomes, exerkines, tumor microenvironment

## Abstract

There are a lot of evidences on the beneficial effects mediated by exercise on the prevention of not communicable diseases (NCDs) including different type of cancer. The production of circulating exerkines transported in exosomes represents a novel pathway activated by exercise. However, the biological mechanisms that could explain the role of exosomes in cancer prevention have been not fully elucidated. The aim of this mini-review is to provide an update on the biological mechanisms bringing the release of muscle-derived exosomes during exercise and cancer prevention.

## Introduction

It is well established that regular engagement in physical activity reduces the incidence of many chronic non-communicable diseases, including hypertension, coronary disease, obesity, type 2 diabetes and cancer (Pedersen and Saltin, 2015; Maridaki et al., 2020; Papadopetraki et al., 2022; Mohr et al., 2023). Current research fully agrees that regular exercise can induce several adaptations including an improvement in: body composition with reduced adiposity, especially in the abdominal region; a greater efficiency in glucose homeostasis and insulin sensitivity (Pedersen and Saltin, 2015; Hoffmann and Weigert, 2017); an improvement in plasma lipoprotein profile; a reduction of systemic inflammation, with enhanced immune response (Franczyk et al., 2023).

The health effects of exercise are largely ascribed to the secretion of a great number of circulating muscle-derived factors released in response to the exercise, namely, myokines. In fact, the skeletal muscle, in response to physical exercise, releases hundreds of secretory products, including proteins, microRNAs (miRNAs) and cytokines into the circulation (Di Felice et al., 2020). Myokines are expressed, produced and released by muscle fibers, exerting autocrine, paracrine or endocrine effects (Severinsen and Pedersen, 2020).

Exercise represents the most important stimulus for myokine release, which represent novel therapeutic targets to counteract both muscular and non-muscular diseases. Recent studies also attributed to myokines a key role in muscle regeneration, a process linked to inflammation that affects many chronic diseases (Di Felice et al., 2022). The latter hypothesis is supported by novel evidences indicating that physical exercise induces the increase of telocytes, a population of stromal cells, in skeletal muscle interstitium, which appears to promote regenerative mechanisms and to support local stem cell differentiation in exercised rodents (Ravalli et al., 2021).

Furthermore, regular exercise promotes the release of circulating small extracellular membranous vesicles, containing myokines, from muscle cells. The mechanisms through which myokines produced during exercise are conveyed in exosomes and involved in cross-talk between organs are topics of great interest (Di Felice et al., 2020).

Here, we provide an update on the biological mechanisms bringing the release of muscle-derived exosomes during exercise and cancer prevention.

### Myokines, exerkines and exosomes

There are about 600 myokines that are regulated in response to muscle contraction (Di Felice et al., 2020). IL-6 was the first described myokine with anti-inflammatory properties in mammals (Pedersen et al., 2003). Some of other myokines are: brain-derived neurotrophic factor (BDNF), angiopoietin-like 4 (ANGPTL4), BAIBA (β-aminoisobutyric acid, a non-protein amino acid), fibroblast growth factor 21 (FGF-21), chemokine (C–C motif) ligand-2 (CCL-2) (also called monocyte chemoattractant protein-1 (MCP-1), chemokine (C–X3–C motif) ligand 1 (CX3CL1) (also called fractalkine (FKN)), irisin, leukemia inhibitory factor (LIF), interleukin-6 (IL-6), IL-7, IL-8, IL-15, myostatin, meteorin-like protein (Metrnl), and secreted protein acidic and rich in cysteine (SPARC) (Hoffmann and Weigert, 2017; Severinsen and Pedersen, 2020).

The beneficial effects of myokines include the regulation of energy expenditure, insulin sensitivity, lipolysis, free fatty acid oxidation, adipocyte browning, glycogenolysis, glycogenesis and general metabolism (Severinsen and Pedersen, 2020).

More recently, novel metabolites as mediators of communication between skeletal muscle and other organs have emerged (Severinsen and Pedersen, 2020): the already mentioned “exerkines” (Safdar et al., 2016; Safdar and Tarnopolsky, 2018). Many studies identified different circulating factors released with the exercise and derived from other organs such as heart (cardiokines), liver (hepatokines), white adipose tissue (WAT; adipokines), brown adipose tissue (BAT; baptokines) and the nervous system (neurokines) (Chow et al., 2022). Exerkines thus consist of a broad range of signalling molecules, including cytokines, nucleic acids (microRNA, mRNA and mitochondrial DNA), lipids and metabolites, which are driven by exosomes and play a key role in the cross-talk between organs during exercise (Vechetti et al., 2021).

**FIGURE 1:**
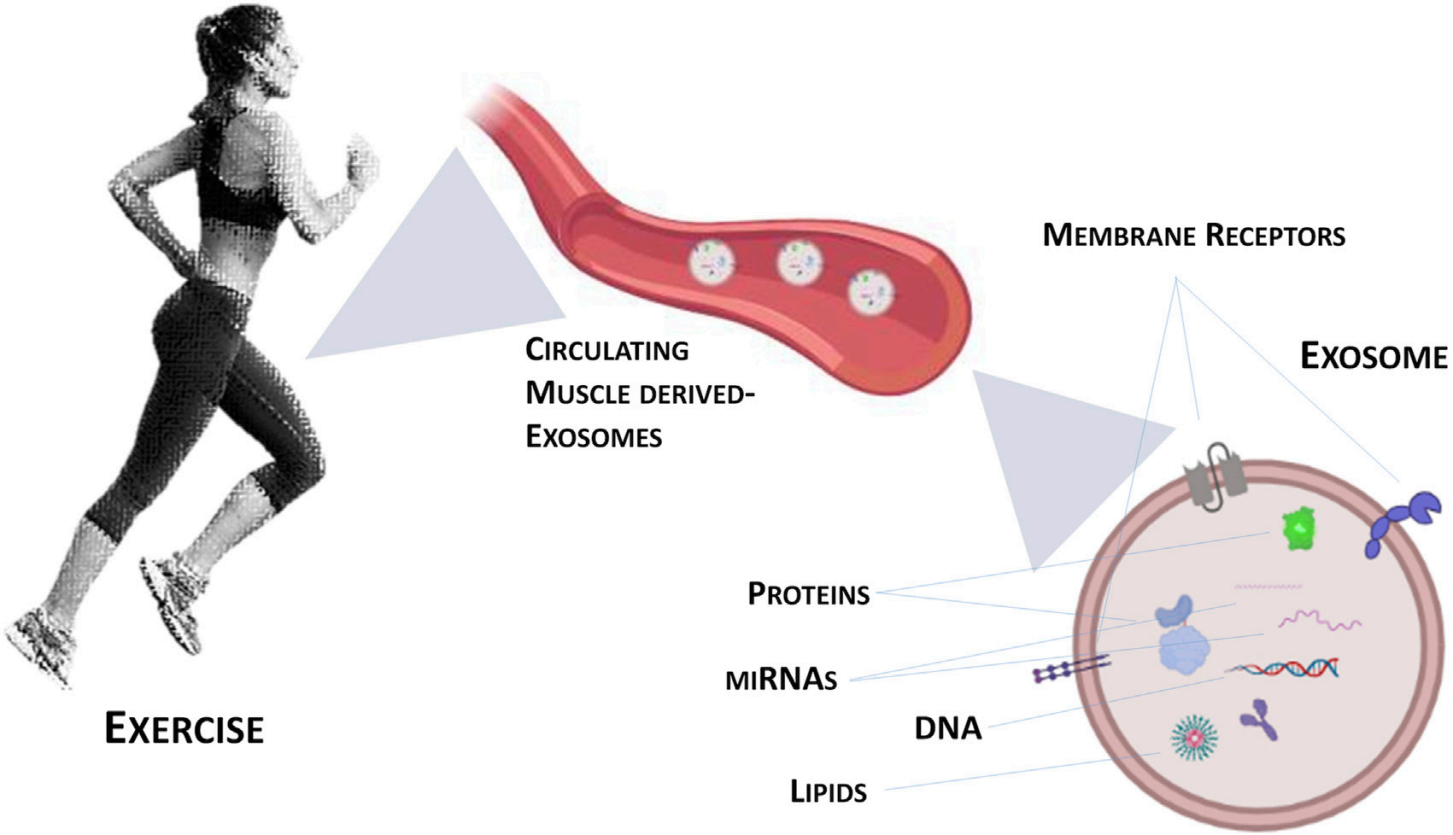
Graphical representation of exercise-induced exosomes released from contracting skeletal muscle.

Exosomes are one type of Extracellular Vesicles (EVs) that differ among them for size, tissue of origin, biochemical composition and density. Through ultracentrifugation, it is possible to classify EVs into large, medium and small size (Trovato et al., 2019). Moreover, EVs are divided in “ectosomes” or “exosomes” based on the biogenesis pathway, i.e., release after the fusion of multivesicular bodies with the plasma membrane or production of micro-vesicles by outward budding of the plasma membrane (Yáñez-Mó et al., 2015; Trovato et al., 2019; Di Felice et al., 2020). Exosomes and their biogenesis represent a protein quality control mechanism, since their release produces a remodelling of the extracellular matrix and the communication among cells (Pegtel and Gould, 2019; Rong et al., 2020). Recent research on exosomes focuses on their ability to deliver complex signals through the engagement and clustering of specific receptors on the cell surface (Dai et al., 2020; Benjamin-Davalos et al., 2021). Guescini and colleagues were the first to demonstrate that skeletal muscle cells produce EVs, including exosomes (Guescini et al., 2010). In addition to proteins, myokines and cytokines, there are other molecules whose production is induced by exercise: miRNAs (see Figure 1). In fact, it has been observed that after exercise the expression of different miRNAs was increased in exosomes (Estébanez et al., 2021) as miR-1 in response to acute cycling exercise (D’Souza et al., 2018) or miR-1, miR-133a and b, miR-206, miR-208a, miR-499 after chronic exercise (Yin et al., 2019). Similarly, other studies evidenced the increase of exosomes circulating-miRNAs as a consequence of different type of exercise (Muroya et al., 2015; Hou et al., 2019).

### Exercise and cancer

In recent years, growing interest on the role of physical exercise in preventing cancer emerged. Approximately 30%–40% of cancer can be prevented by lifestyle modification, including physical exercise, diet and environmental factors (Huang et al., 2022). However, the effect of exercise in reducing cancer risk depends from intensity and frequency (Wang and Zhou, 2021). Most evidences on the effect of exercise on breast cancer has been provided: the incidence was reduced in fertile women who performed high-intensity exercise for at least 3–5 h/week, even in postmenopausal women (Magnè et al., 2011; Desnoyers et al., 2016; Orlandella et al., 2021).

Regular exercise, together with a healthy lifestyle, has a positive impact on the incidence of colorectal cancer: many studies indicated that the risk of this cancer was reduced of 19% in subjects with higher levels of fitness (Liu et al., 2016), and similarly, on gastric cancer incidence, where it was reduced of 19% in trained subjects (Psaltopoulou et al., 2016). In smokers, the risk of lung cancer was lower in exercised subjects, even if this benefit disappeared in non-smokers (Schmid et al., 2016). Exercise also affects the IGF-1 signaling pathway involved in cancer proliferation/survival, expecially in different epithelial tumors thus explaining, at least in part, the molecular mechanisms underlying cancer prevention (Cevenini et al., 2018). Conversely, limited evidences in other types of cancers (such as blood, pancreatic, ovarian) regarding the effects of exercise on prevention and progression of cancer were reported (Wang and Zhou, 2021).

### Exosomes and cachexia

Present in up to 80% of cancer patients, cachexia is a complex metabolic syndrome characterized by significant loss of muscle mass (up to 75% of skeletal muscle mass) with poor quality of life and decreased survival (Tisdale, 2010). Cachexia is considered the immediate cause of death in 20%–50% of all cancer patients (Marinho et al., 2018). Despite its clinical relevance, this syndrome is underdiagnosed and not yet fully elucidated.

High levels of circulating pro-inflammatory cytokines associated with tumor cachexia represent a potential target of exercise. Exercise, in fact, represents a non-pharmacological therapeutic strategy, in synergy with anticancer therapy, to counteract systemic inflammation through the production and secretion of anti-inflammatory myokines, thus promoting the reduction of muscle wasting and the improvement of response rates and survival (Leal et al., 2021).

Furthermore, recent studies demonstrated that one of the mechanisms that may be involved in the transduction of inflammatory signals and the activation of the catabolic state in muscle is linked to exosomes containing miRNAs and myomiRs. He and colleagues evidenced that lung and pancreatic cancer cells secrete exosomes containing miR-21 that, once transported in the bloodstream, is able to induce apoptosis of muscle cells (He et al., 2014). More recently, Zhang and colleagues (Zhang et al., 2017) demonstrated that HSP70 and HSP90 proteins, present in the membrane of exosomes, are released by cancer cells thus inducing muscle wasting in cancer cachexia models. Conversely, Hudson and colleagues showed that miR-182, present in exosomes, counteracts the role of Foxo3 in inducing atrophy in the skeletal muscle (Hudson et al., 2014).

Interestingly, recent evidences demonstrated that exercise could increase cytoprotective proteins expression, counteracting muscle atrophy. In particular, exercise improves the expression of Hsp60 protein, associated to mitochondrial biogenesis and oxidative capacity of muscle cells (Di Felice et al., 2022). Similarly, Morton and colleagues found significantly increased (25%) Hsp60 expression levels in the *vastus lateralis* muscle of trained compared to sedentary individuals (Morton et al., 2008); moreover, a significant release of Hsp60-bearing exosomes was found in the blood of BALB/c mice subjected to a 6-week training program compared to sedentary animals (Campanella et al., 2008). Recently, a potential anti-cachexia drug based on Hsp60-containing nanovesicles has been proposed, which could mimic the beneficial effects of exercise by improving patient survival and quality of life (Di Felice et al., 2022).

### Exercise-induced exosomes and cancer

Although several mechanisms have been hypothesized as crucially related to the anti-cancer benefits of exercise, the exact mechanism by which exercise may produce this effect remains unclear (Magnè et al., 2011; Goncalves et al., 2014; Desnoyers et al., 2016; Reis et al., 2017; Wang and Zhou, 2021). It has been demonstrated that exercise, through the circulating muscle derived-exosomes, secretes more than 300 molecules such as proteins, myokines, miRNAs and glycolytic enzymes (Whitham et al., 2018). It can act as a tumour suppressor, both by influencing several distinctive features of cancer cells (Hojman et al., 2011; Ruiz-Casado et al., 2017) and by inducing changes in the metabolic activity of tumour cells (Pedersen and Saltin, 2015). In fact, exercise can affect cancer metabolism and anaerobic glycolysis, strongly enhanced in cancer cells: these effects on tumor metabolism represent an useful tool to better understand cancer biology and to develop therapies targeting cancer energy metabolism (Vulczak and Alberici, 2022).

Muscle-derived exosomes appear to directly interact with tumour cells by altering their structure, as well as by modifying the function of tumour-infiltrating immune cells, thereby influencing the growth rate of cancer cells (Sadovska et al., 2021). Recent data provide novel evidence on the effects of muscle-derived exosomes on delay prostate cancer progression and metastasis, acting on several physiological processes including protein folding, energy metabolism and regulation of immune responses triggered by exercise (Sadovska et al., 2021). Furthermore, a pattern of several miRNAs in the urinary exosomes including miR-21, miR-451 and miR-636 has been recently identified as non-invasive prognostic biomarker for prostate cancer (Shin et al., 2021; Zhang et al., 2021). Finally, Bryant and colleagues demonstrated that several miRNAs, such as miR-107, miR-130b, miR-141, were increased in circulating exosomes from prostate cancer patients compared to healthy individuals, reinforcing the relevance of molecules carried by exosomes as prognostic markers for prostate cancer (Bryant et al., 2012).

Exercise-induced miRNAs are closely linked to the *Hippo Tumor Suppressor Pathway*, whose activation, through the inhibition of two homologous transcription factors, the *Yes-Associated Protein* (YAP) and Transcriptional Co-activator with *PDZ-binding Motif* (TAZ), block target genes, involved in cancer cell proliferation and survival (Badouel and McNeill, 2011; Yu et al., 2013). Du and colleagues recently demonstrated that miR-223-3p transcription levels were increased in breast cancer cells. The inhibition of miR-223-3p transcription reduces proliferation, migration and invasion of breast cancer cells, through the *Hippo/Yap* signaling pathway (Du et al., 2021). Furthermore, Dethlefsen and colleagues demonstrated that catecholamines induced by exercise could activate the *Hippo Tumor Suppressor Pathway,* thus reducing the risk of breast cancer development (Dethlefsen et al., 2017).

Furthermore, circulating muscle-derived factors produced during exercise can counteract tumorigenesis and cancer progression by influencing the tumor microenvironment constituted by different cell types, mechanical and chemical stressors and humoral factors (Koelwyn et al., 2017). The interaction of all these components greatly affects cancer cells and, subsequently, the growth rate of tumor. To data, few studies focused on the exercise and tumor microenvironment adaptations. Koelwyn and colleagues pointed out that exercise affects tumor microenvironment through several mechanisms such as tumor perfusion, vascularization, hypoxia and immune response, playing a key role in cancer suppression (Koelwyn et al., 2017).

**FIGURE 2:**
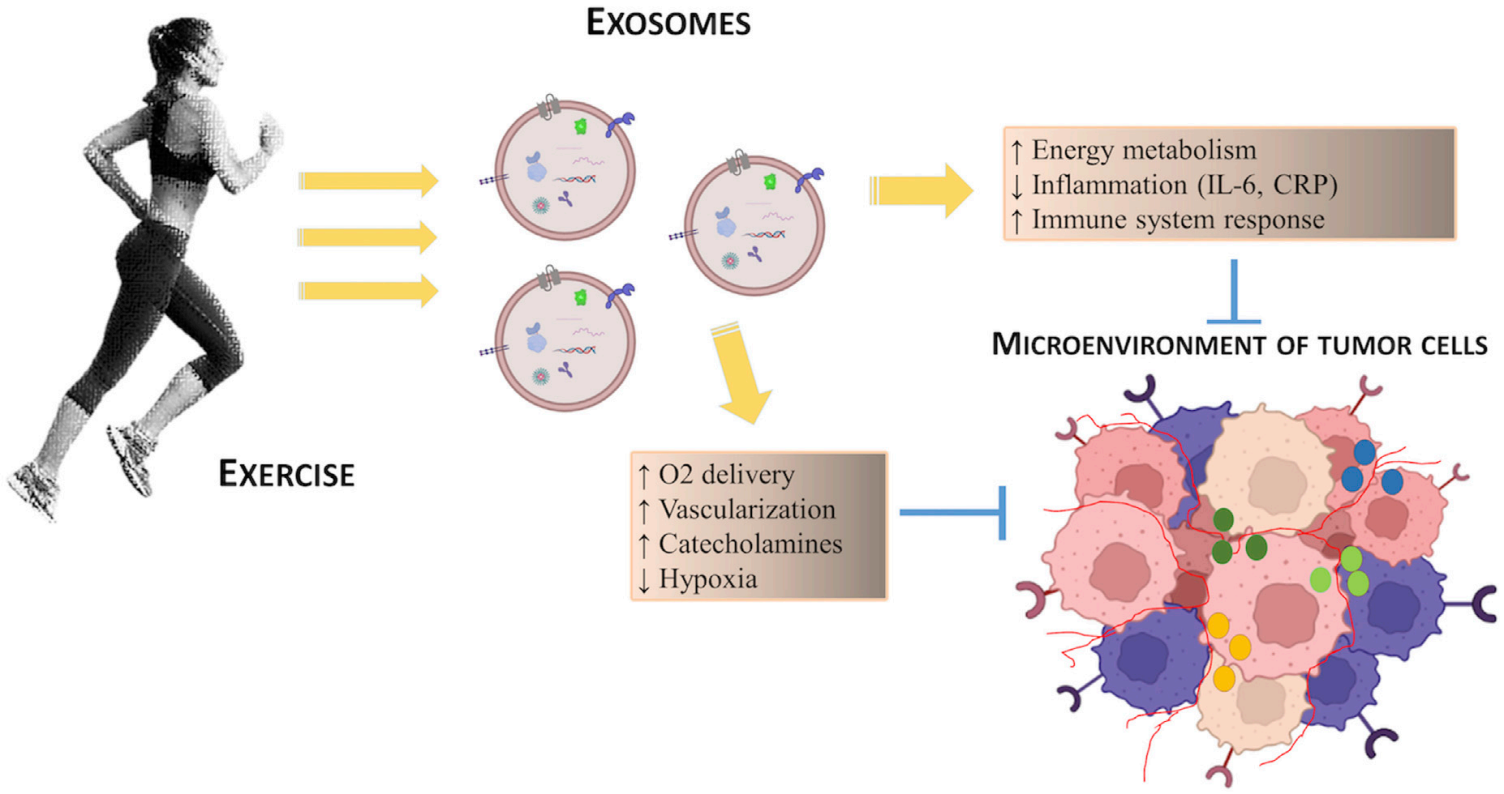
Schematic representation of the potential effects of exercise on the regulation of tumor microenvironment.

Regular exercise stimulates cross-talk between organs through the secretion of hormones, cytokines and growth factors from various tissues, including skeletal muscle, which promotes many adaptations, including: enhancing energy metabolism through the availability of nutrients, and the modulation of growth factors such as insulin and IGF-1 which promote cell proliferation; reducing inflammation by decreasing the circulating levels of cytokines such as IL-6 and C-reactive protein (CRP) with evident pro-tumorigenic action (see Figure 2). These exercise-induced adaptations also modify key regulatory mechanisms of tumor microenvironment, such as angiogenesis, immune regulation and metabolism, thus having a cumulative antitumorigenic effect. Furthermore, during exercise, blood flow is redirected to active skeletal muscle with surprisingly increased tumor blood perfusion and reduced tumor hypoxia: this represents an alternative mechanism of exercise regulation on the tumor microenvironment (Koelwyn et al., 2017).

Finally, regular exercise, through exercise-induced circulating molecules, also modifies the immune-system response as demonstrated by many studies that highlighted the role of miR-486-5p in regulating the breast cancer microenvironment, through increased recognition of tumor cells by cytotoxic T lymphocytes and natural killer cells in patients with breast cancer (ElKhouly et al., 2020; Siqueira et al., 2021, 2023).

## Discussion

The relevance of exosomes as carriers for exerkines and their role in the prevention of NCDs and cancer, including cachexia, have pointed out. More evidences have been provided supporting the hypothesis that skeletal muscle, during contraction, secretes not only myokines and cytokines, already widely discussed, but also miRNAs and other bioactive molecules (including DNA and proteins), which are secreted in exosomes whose concentration is regulated by type and intensity of the exercise (Fruhbeis et al., 2015; Whitham et al., 2018; Nielsen et al., 2019; Rigamonti et al., 2020).

These transport systems involved in cell-to-cell communication require a broad spectrum of mechanisms that allow signal transduction; all these systems are not well described in skeletal muscle cells, and therefore the detailed study of their function will be of great interest, as they can mediate the effects induced by exercise on the regulation of cancer (Darkwa et al., 2021).

In this context, physical exercise, through exerkines, seems to have multitarget actions, which directly or indirectly, can act on tumor microenvironment by modifying the anti-cancer immune response, promoting vascularization, hypoxia and tumor perfusion and modifying energy metabolism in tumor cells.

## Conclusion

It therefore appears clear that myokines induced by exercise and transported in exosomes can induce systemic effects, so that they can be considered novel molecular targets to prevent the onset of cancer and/or to delay the development of disease.

Future studies will be needed to gain insight into the intricate molecular and exercise-induced regulatory mechanisms that control the release of both exerkines and exosomes and their role in regulating cancer development. Moreover, further insights are required to develop tailored exercise protocols useful to counteract cancer progression and cachexia and promote a better quality of life in cancer survivors.
